# Editorial: Discovery of digital biomarkers in the background of big data and advanced machine learning

**DOI:** 10.3389/fphys.2023.1239219

**Published:** 2023-07-06

**Authors:** Ming Huang, Wenxi Chen, Toru Nakamura, Yuichi Kimura

**Affiliations:** ^1^ School of Data Science, Nagoya City University, Nagoya, Japan; ^2^ Graduate School of Science and Technology, Nara Institute of Science and Technology, Ikoma, Japan; ^3^ Division of Information Systems, The University of Aizu, Aizuwakamatsu, Japan; ^4^ Institute for Datability Science, Osaka University, Suita, Japan; ^5^ Faculty of Informatics, Kindai University, Higashiosaka, Japan; ^6^ Cyber Informatics Research Institute, Kindai University, Higashiosaka, Japan

**Keywords:** digital health, digital biomarkers, medical images, biosignal, advanced machine learning, domain adaption

There has been an explosive growth of data generated from medical and healthcare devices over the past decade. A very important task in the computational area is to extract reliable surrogates of conventional physiological markers. For example, heart rate variability has been shown indicative of the autonomic nervous system. Recently, promising surrogates started to emerge from the complicated transformation from a bundle of signals or medical images based on artificial intelligence and big data analytics. Digital biomarkers depend largely on digital technologies, in addition to the in-lab and in-hospital medical devices, personal devices such as wearables, smartphones, and other sensors that collect data on an individual’s physiology, behavior, and environment are useful in digital biomarker generation. The concept of digital biomarkers is related to the machine learning application to physiology and medicine, the latent features of a deep learning model could be regarded as a digital marker. Additionally, it is anticipated that important added values will be extracted from convenient devices on a daily basis, such as being more sensitive than a conventional intervention marker. Of note, considering the personal difference that exists in almost all kinds of biosignals, a potential marker should be statistically significant.

At present, digital biomarkers are still in the early stages of development, and there is much research being conducted to establish their validity and reliability. Scientists are carrying out large-scale studies to collect and analyze vast amounts of data, utilizing advanced machine-learning algorithms to identify patterns and predict potential disease outcomes. Digital biomarkers are being developed for a wide range of diseases, including neurological disorders, cardiovascular diseases, and mental health conditions. These biomarkers can potentially transform healthcare, providing clinicians with real-time data to make more accurate diagnoses and personalized treatment plans. Despite the promise of digital biomarkers, there are challenges to their validation and implementation, such as model generalization, privacy concerns, *etc.,* In this Research Topic, we have received a number of articles and have 5 of them successfully published. Of these excellent articles, two focused on extracting useful information from medical images to aid in diagnosis, while the other three aimed to generate useful information for healthcare and its possible application in diagnosis.

Pre-trained deep learning models are becoming increasingly valuable diagnostic tools as deep learning technologies become more integrated into radiology. In the article titled “Uneven Index: A Digital Biomarker to Prompt Demodex Blepharitis Based on Deep Learning”, a validated pre-trained model based on 50-layer ResNet was used to extract the meibomian glands (MGs) in the eyelids Liu et al. This automated segmentation conduces to the extraction of morphology indexes of the MGs, and ultimately the formation of the new uneven index, a new index that measures the unevenness of MG atrophy. Due to its ability to capture overall changes in MG morphology, the uneven index may serve as a useful digital biomarker for diagnosing Demodex blepharitis (AUC of ROC curve = 0.82).

In the article titled “Contrastive learning and subtyping of post-COVID-19 lung computed tomography images”, Using a lung volume transform to learn latent features from CT scans of normal and infected lungs, the authors demonstrate how a contrastive learning model can be used to automatically identify post-COVID-19 patients from normal individuals with an averaged accuracy of 90.00% on the testing dataset Li et al. The latent features learned by the model are further analyzed using k-means clustering, from which the relation between the clusters and the clinical phenotypes (C1: increased air-trapping; C2: decreased lung volume) can be seen.

Since biosignals, such as electrocardiograms (ECGs), can be measured much more easily than medical images, latent features learned from the biosignals can be applied to daily healthcare practices as well. The authors created a deep learning model using convolutional layers to classify 6 levels of fatigue in their article titled “Exercise Fatigue Diagnosis Method Based on Short-Time Fourier Transform and Convolutional Neural Network”. The application of a convolutional neural network to the frequency domain of ECG signals yields accurate outcomes in determining fatigue levels (accuracy = 97.70%) Zhu et al. Nonetheless, it remains uncertain whether validation is performed on an individual basis. Given that data acquisition and model integration are uncomplicated, this approach can be utilized for instant personal workout guidance with minimal lag time.

The article titled “EEG-based real-time diagnostic system with developed dynamic 2TEMD and dynamic ApEn algorithms” proposed a variant of the empirical model decomposition to compute EEG energy and a variant of approximate entropy to compute the EEG complexity Zhang et al. The authors demonstrate the usefulness of the suggested metrics in determining brain death (Accuracy of D-2TEMD: 97.0%, D-ApEn: 94.0%). Since energy and complexity are two important metrics in EEG analysis, it is expected that these two metrics can be applied to other tasks such as sleep stages analysis.

When creating a pipeline to extract important features or metrics for a specific task, it is crucial to make sure that these features or metrics can be used effectively in different situations, like with various subject populations and measuring systems. To ensure their usefulness in different scenarios, it is important to validate them across multiple datasets and measuring systems. This is a good practice when developing computational models. In the article “Training a Pipeline for Detecting Atrial Fibrillation using Photoplethysmography Signal”, the authors optimized a transfer learning pipeline that fine-tunes an AF classifier trained with ECG signal to create a new classifier for PPG signal input. By using an advanced deep learning model, the optimized pipeline has achieved a performance level of 0.91 recall and 0.79 precision Kudo et al. This highlights the importance of standardizing the validation process for biomedical purposes.

The field of digital health holds immense potential to enhance healthcare outcomes through ongoing research and development efforts that aim to leverage the power of digital technologies. digital biomarkers are vital in the field of digital health as it facilitates personalized healthcare and preventive medicine, leading to a significant transformation in the medical practice. With the advancement of AI technology and the Research Topic of more data, we can anticipate a rise in task-specific applications and the emergence of more significant digital biomarkers.

